# Relative Stability of Core Groups in Pollination Networks in a Biodiversity Hotspot over Four Years

**DOI:** 10.1371/journal.pone.0032663

**Published:** 2012-03-08

**Authors:** Qiang Fang, Shuang-Quan Huang

**Affiliations:** College of Life Sciences, Wuhan University, Wuhan, China; University of Northampton, United Kingdom

## Abstract

Plants and their pollinators form pollination networks integral to the evolution and persistence of species in communities. Previous studies suggest that pollination network structure remains nested while network composition is highly dynamic. However, little is known about temporal variation in the structure and function of plant-pollinator networks, especially in species-rich communities where the strength of pollinator competition is predicted to be high. Here we quantify temporal variation of pollination networks over four consecutive years in an alpine meadow in the Hengduan Mountains biodiversity hotspot in China. We found that ranked positions and idiosyncratic temperatures of both plants and pollinators were more conservative between consecutive years than in non-consecutive years. Although network compositions exhibited high turnover, generalized core groups – decomposed by a *k*-core algorithm – were much more stable than peripheral groups. Given the high rate of turnover observed, we suggest that identical plants and pollinators that persist for at least two successive years sustain pollination services at the community level. Our data do not support theoretical predictions of a high proportion of specialized links within species-rich communities. Plants were relatively specialized, exhibiting less variability in pollinator composition at pollinator functional group level than at the species level. Both specialized and generalized plants experienced narrow variation in functional pollinator groups. The dynamic nature of pollination networks in the alpine meadow demonstrates the potential for networks to mitigate the effects of fluctuations in species composition in a high biodiversity area.

## Introduction

Community studies have shown that the network structure of plant-pollinator interactions is largely asymmetrical — the partners of specialists tend to also interact with generalists [1]–[5]. Within a community some plants are pollinated by a large proportion of the available flower visitors (generalization) while others are pollinated by a relatively smaller proportion (specialization) [6], suggesting that ecological generalization predominates [7]–[10]. A relatively unexplored question is the extent to which ecological generalization is stable across multiple flowering seasons through the persistence of specific linkages.

Documenting network stability is essential for an understanding of how specialized plants evolve in communities dominated by generalized pollination networks. If pollinator-mediated selection is one major force driving the evolution of flowers, the selective role of the pollinators could be diminished if plant-pollinator interactions are highly variable across years [11], [12]. Recent multi-year studies, including four conducted in Europe [13]–[16] and two in North America [17], [18], show that large temporal differences in network structure and species interactions are largely attributable to species turnover across years and flexibility in interactions with new partners; but the basic topological properties of plant-pollinator networks remain unchanged [19]. For example, in a montane meadow community from southern California 36% of plant species and 18% of pollinator species were shown to have specialized links with at least one mutualistic partner across three summers [17]. In a scrub community in Greece, 53% of the plant species and 21% of pollinators, but only 5% of species interactions, were observed across four consecutive years [14].

However, our understanding of temporal variation in plant-pollinator interactions is currently limited, given that we know little about which kinds of species are likely to turn over, particularly in species-rich communities. A distinct property of pollination networks is that they are highly nested; that is, most species interact with hierarchical subsets of generalist partner species [6]. In a nested pattern, it has been suggested that generalists are key components to maintain network stability and to resist the susceptibility to extinction of specialists [20]. Although generalists contribute more to network stability than specialists, field investigations have shown that many plant or pollinator species link to only one partner per year and tended to be ecologically specialized, although they may be considered generalists in that they interact with different partner species in other years [14]. Furthermore, all community-level surveys must be considered to be samples of the true diversity and hence apparently specialist plants may be pollinated by other pollinators which were not detected in the surveys. To explore the dynamic nature of the plant-pollinator network, we investigated temporal stability in an alpine meadow over four years using a *k*-core analysis. The *k*-core algorithms can determine which plants and pollinators composed a “*k*”-core group (*k* is an integer), depending on link number and partners' link quality. This *k*-core analysis is a bottom-up method to separate the network into subgroups [21]. It has been used to classify protein positions in protein networks to show the evolutionary trend in co-occurrence networks from single cells to multicellular eukaryotes [22] and also to identify subgroups of taxa in plant-pollinator networks [5].

We investigated plant-pollinator interactions in a species-rich alpine meadow of the Hengduan Mountains biodiversity hotspot in China [23]. Plants in species-rich communities may be pollinated by more specialized pollinators than those in species-poor communities. Although there was a positive relationship between plant species number and pollinator species number, studies suggested that high species diversity could reduce pollinator niche overlap, allowing pollinators to focus on specific plant species for nectar or pollen resources [24], [25]. However, Ollerton et al. (2003) found significant pollinator overlap between asclepiad species in a species-rich grassland in South Africa [2]. Previous analysis suggested that plants in species-rich communities may be more prone to pollen limitation than those in less species-rich areas because of interspecific competition for pollinators [26]. Based on null models, a larger proportion of extreme specialists and generalists are both expected to appear in a species-rich community as the number of interacting species increases [9]. Therefore, plants in biodiversity hotspots are likely to experience a higher risk of extinction and/or to specialize on certain pollinators [26].

Plants may be pollinated by a taxonomically diverse group of pollinators that all function in a similar way [27]; those pollinators sharing similar behavior or flower preference are categorized as being in the same functional group. For example, *Pedicularis* species in the study community were generalized – linked to several pollinator species – but they are specialized at the functional group level because they are only pollinated by bumble bees. It has been suggested that variation in pollinator composition can not be assumed to reflect coevolutionary relationships between plants and pollinators unless one considers the similar behavior and flower preference of members of the same pollinator functional group [28], [29]. Thus, one would expect that temporal fluctuation in pollination networks could be cushioned if one lost pollinator species can be replaced by another from the same pollinator functional group. An estimate of the temporal stability of functional groups in pollination networks may provide insights into the difference between ecological and evolutionary specialization [28], [30]. However, the temporal variation of functional pollinator groups has not been evaluated at a whole community level [19], [28], [31].

Studies of network stability have been conducted in Europe and North America, in relatively species-poor communities, ranging from 7 to 39 plant species and 23 to 597 pollinator species [13], [15]–[18]. Only two investigations in large areas recorded over 100 plant species in Europe [14], [15]. Community studies of the pollination network in the Hengduan Mountains biodiversity hotspot permit us to examine whether ecological specialization and/or generalization tends to be higher in species-rich communities through a comparison to previous studies in species-poor communities, as predicted by theoretical models [26]. Specifically, we addressed the following questions. (a) How great is inter-annual variation in the alpine meadow pollination network? Given that we have four years' data, we asked whether the network was more similar between consecutive years than non-consecutive years. (b) Which kinds of species are likely to turn over in pollination networks with relatively stable structures, the inner or periphery species? We examine whether there is a certain pattern in species turnover by *k*-core decomposition. (c) Is the variation in pollinator composition across years similar in ecologically generalized and specialized plants at species and functional group level?

## Materials and Methods

### Study community

To document plant-pollinator interactions (links), we investigated a natural plant community at Shangri-La Alpine Botanical Garden (27°54′5″N, 99°38′17″E, 3300 m to 3350 m a.s.l.), Yunnan Province, China, over four years. The field station is located southeast of the Hengduan Mountains, one of the richest biodiversity hotspots in the world [23]. Pollinator observations were conducted within an 800×250 m^2^ area of sloping meadow. Approximately 20% of the field was moist meadow, with species of *Primula* and *Trollius* dominant. *Aster*, *Astragalus pullus*, *Halenia elliptica*, *Potentilla lancinata* and several Apiaceae species dominated the dry meadow plots. *Pedicularis* species were common in both types of meadow. All observed species are listed in Supporting Information S1. All necessary permits were obtained for the described field studies. The investigations comply with the current laws and policy of biodiversity conservation in China.

### Sample procedure

Field work was conducted during peak flowering time: from July 10 to August 26 in 2007, July 13 to August 24 in 2008, July 10 to August 27 in 2009 and July 10 to August 29 in 2010. Pollinator visitations were normally recorded during the daytime from 9:00 to 18:00. The peak period of insect activity was from 11:00 to 16:00. Outside of this time period, low temperatures limit pollinator activity, except by moths. In 2007 flower-visiting insects were observed along a pair of transects, taking 70–90 min per round. Pollinator visits to nearby plant species were recorded. In 2008, 2009 and 2010, we monitored all flowers of all species observable from a fixed observation plot for a period of 30 minutes. Some observation sites were specifically chosen to include rare species to ensure they were adequately sampled. We recorded a pollination visit if an insect contacted the plant's reproductive structures while actively searching for pollen and/or nectar [32]. Using this observation procedure, each plant species was observed for a minimum of 2 hours. In total, we observed for 100, 135, 134 and 132 hours on clear days in 2007, 2008, 2009 and 2010, respectively. Common and easily identifiable pollinator species were identified and released in the field and unknown visitors were collected and sent to the Institute of Zoology, CAS, China for further identification to species or higher level groups. Pollinators of each plant species in four years are listed in Supporting Information S1.

### Network parameters

We created a binary matrix representing observed versus not-observed interactions between plants and pollinators (A×P). In binary matrices, “1” represents an observed visit, and “0” represents no visit. Summary statistics for each matrix were then calculated, including the total number of flower visits recorded, number of plant-pollinator links, and mean and maximum number of links per plant and per pollinator species. We separated the pollinators into 9 functional groups (bee, bumblebee, beefly, fly, hoverfly, butterfly, beetle, ant and other) based on similar behavior or flower preference [29], [33].

### Data analysis

To measure nestedness, we analyzed the A×P matrix with the ANINHADO 3.0 program for both System temperature (T) and NODF value (http://www.guimaraes.bio.br/softwares.html) [34]. System temperature (T), is a measure of disorder of the network. For a perfectly nested matrix (T = 0), each species is linked to a subset of the partners of the next less specialized species, and the most generalized plant and pollinator species are each linked to all partner species in the matrix. The statistical significance of T is calculated by comparing the actual matrix temperature to the temperatures of 1000 matrices generated by Monte Carlo randomizations. T was subsequently converted to the nestedness index N (N = (100−T)/100), which ranges from 0, when the network is randomly organized, to 1, when it is perfectly nested [1]. The NODF (nestedness metric based on overlap and decreasing fill) approach is a gap based metric compared to system temperature. It accounts for paired overlap and decreasing fill of the matrix and claims to reduce type I statistical errors compared with T [35]. NODF scales from 0 to 100, where 100 represents maximum nestedness.

In order to compare species' roles within the plant-pollinator network among years, we calculated each species' ranked position and idiosyncratic temperature within each of the four years, then compared values among years using Spearman's rank correlation [cf. 17]. Calculations were based on the binary matrices (see Supporting Information S1). A pollinator or plant species' rank refers to its location along the columns or rows of the A×P matrix when the matrix is arranged to exhibit maximum nestedness [34]. Species with lower ranks tend to be more generalized, but the most linked node is not necessarily first in a maximum nestedness network. For comparisons among years, rank values were standardized by dividing the rank of a species in a year by the total number of pollinators or plants observed in that year. A species' idiosyncratic temperature (IT) measures how the species' actual link pattern in the maximally nested network deviates from the expected pattern if the network were to exhibit perfect nestedness [36]. Thus, species with higher proportions of unexpected links have higher ITs.

We used graph theoretical algorithms (*k*-core) to determine which plants and pollinators composed inner and periphery groups. We defined core groups as those that form link nodes which are persistent across years. Plants and pollinators are represented as nodes in the graph and their interactions are represented as edges connecting the nodes. As a bottom-up method, the *k*-core algorithms separate the network into subgroups. A subset of interconnected species among which each species has at least k interactions form a *k*-core [37]. Species with more links, being more generalized, will be included in higher subgroups. The subgroup with the highest *k* core grade is the innermost core of the network, and the subgroup with *k* equal to 1 is the most peripheral core of the network. Analyses were performed in UCINET 6.24 (http://www.analytictech.com) [21].

We explored whether turnover rates differed between inner and peripheral groups from year to year. Due to the size of the pollination networks, the plants and pollinators were separated by *k*-core grade into 4 subgroups in 2007, 8 subgroups in 2008, 7 in 2009 and 6 in 2010. For comparison we standardized the subgroup number. The 8 subgroups in 2008 were merged into 4 subgroups (the number of groups identified for the smaller 2007 data set). For the 7 subgroups in 2009, the first and last subgroups were separated, the second and third subgroups were merged and the forth, fifth and sixth were merged. For the 6 subgroups in 2010, the first and last subgroups were separated and other four subgroups were merged into 2 subgroups. Following this procedure, we could standardize species number of each subgroup to the other three years. Then we calculated turnover rates for both plants and pollinators within each group between each pair of years. We tested whether turnover rates for plants or pollinators changed with increasing generalization (higher *k*-core grade) using Spearman's rank correlations. Then, we combined the inner half and periphery *k*-core grades to examine whether species of inner grades had less turnover than species in peripheral grades using G-tests. We also tested whether major pollinator functional groups (bee, fly, hoverfly and butterfly) are distributed differently in the inner-periphery pattern. To compare each major pollinator group's distribution between inner and periphery grades, we performed G-tests using total species number of inner and periphery grades as expected numbers. Finally, we investigated whether ecologically generalized plants experienced stable pollinator spectra, by calculating the Bray-Curtis distance (also called the Sorensen distance) of pollinator functional groups for each plant species between all pairs of years to represent variation in pollinator composition [19], [38]. Species number of each functional group was standardized by dividing by the total pollinator number for each plant in each year. The Bray-Curtis distance ranged from 0, where the plant species had same functional group partition in both years; to 1, where the plant experienced entirely different pollinator groups in the two years.

We used SPSS 13.0 to conduct standard statistical tests. All means are presented ± SD.

## Results

### Dynamic network structure

**Figure 1:**
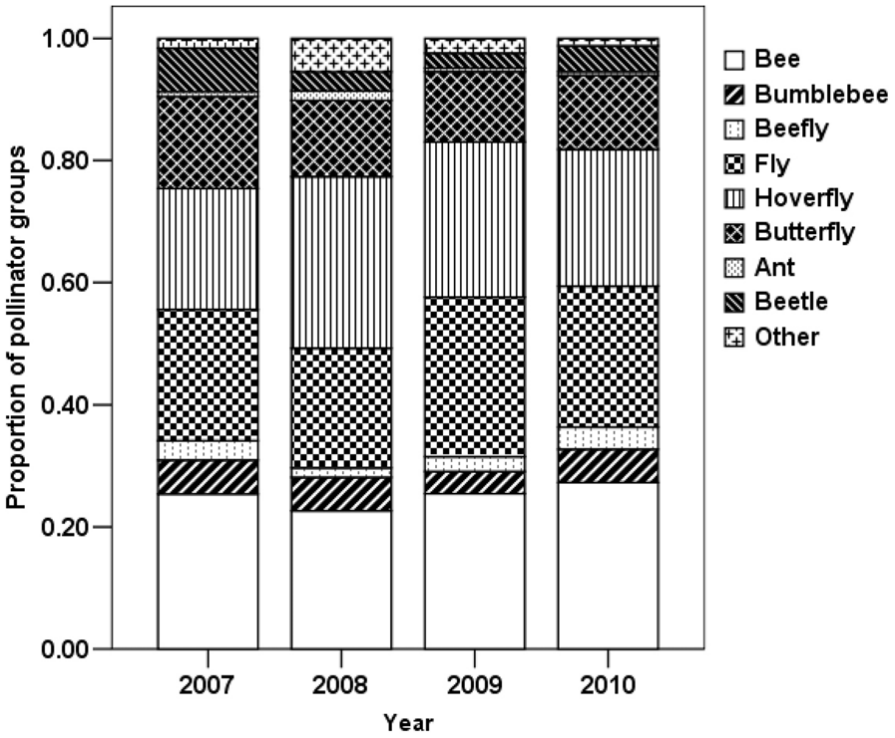
Proportional representation of 9 pollinator functional groups in 4 consecutive years. The 9 groups are bee, bumblebee, beefly, fly, hoverfly, butterfly, ant, beetle and others.

In all four years, plant-pollinator networks were significantly nested (Table 1). Overall, there was little variation in system temperature, with average nestedness of 0.960 (SD = 0.007). The analysis by the NODF algorithm also showed that the networks were significantly nested (Table 1). The turnover rate of links between years (82.1%±4.6%) was greater for pollinators (59.1%±7.3%) than plants (23.1%±10.3%) (Table 2). The species of plants for which we observed visits varied among study years, and there was even greater variation among pollinators (Table 2). Similarity among plant-pollinator links was less than 10% for half of the pairs of years, lower than the similarity of network components (both plants and pollinators) (Table 2). In all four years, *Bombus richardsi* was the most generalized pollinator, which linked to 51%, 66%, 51% and 66% of plants, respectively. Despite the variation in pollinator community composition, the proportional representation of functional groups (Fig. 1) was not significantly different between consecutive years (G test, 2007 vs 2008, G = 8.40, p = 0.40; 2008 vs 2009, G = 4.64, p = 0.80; 2009 vs 2010, G = 4.60, p = 0.80, all df = 8) and also non-consecutive years (G test, 2007 vs 2009, G = 7.24, p = 0.51; 2007 vs 2010, G = 4.32, p = 0.83; 2008 vs 2010, G = 6.44, p = 0.60).

**Table 1:** Parameters of the plant–pollinator networks.

	2007	2008	2009	2010
Plant species (total 138)	79	88	100	108
Plants as % of total species	59.4%	66.2%	75.2%	81.2%
Mean links per plant (± SD)	5.7±7.2	8.9±7.7	8.4±7.1	7.2±5.6
Maximum links in plant	43	45	33	26
Pollinator species (total 347)	126	128	149	149
Pollinators as % of total species	38.1%	38.7%	45.0%	45.0%
Mean links per pollinator (± SD)	3.6±4.8	6.12±8.6	5.7±7.8	5.2±8.4
Maximum links in pollinator	40	58	51	71
Interactions (total 2293)	452	790	847	781
Interactions as % of total interactions	19.7%	34.5%	36.99%	34.1%
Connectance	4.54	7.01	5.67	4.85
Nestedness	0.968^**^	0.952^**^	0.957^**^	0.963^**^
NODF	19.17^**^	25.44^**^	29.43^**^	32.10^**^

Values are given for observations made in each year as well as the entire 4-year study period. Connectance is the percentage of interactions divided by the number of possible interactions; Nestedness is (100−T)/100, where T is the temperature. NODF (Nestedness based on overlap and decreasing fill) values are explained in the text (Methods). (* P<0.05; ** P<0.01).

**Table 2:** Similarity of plants, pollinators and their interactions between each pair of years.

	2007 vs 2008	2007 vs 2009	2007 vs 2010	2008 vs 2009	2008 vs 2010	2009 vs 2010	Four years
Total number of species/interactions observed over the 2 years							
Plants	106	117	124	112	120	117	133
Pollinators	203	221	231	208	226	227	331
Interactions	1140	1209	1166	1572	1411	1460	2293
Number of species/interactions observed in both years							
Plants	61	62	63	76	77	92	54
Pollinators	51	54	42	69	51	71	28
Interactions	102	99	68	164	160	167	31
Jaccard similarity index %							
Plants	57.5	53.0	50.8	67.9	64.2	78.6	40.6
Pollinators	25.1	24.4	18.0	33.2	22.6	31.3	8.5
Interactions	8.9	8.2	5.8	10.4	11.3	11.4	1.4

### Stability of networks

The patterns of plant positions in the nested interaction matrix were quite stable across years. Standardized ranks in plants and pollinators were all positively related between each pair of years (Table 3). In both plants and pollinators, idiosyncratic temperature (IT) was significantly correlated in 3 of 6 pairs of years (Table 3). These findings suggest that the plants and pollinators were in the same position in the nested interaction matrix across years, and between certain years they were interacting with similar partner species but between other years they were not. More positive relations in IT were observed between consecutive years (2/3) than between non-consecutive years (1/3), indicating that the pollination networks were more similar between consecutive years (Table 3).The pollinators and plants appearing in all four years were probably more important for community sustainable compared to the turnover species, because they continuously provided pollination service and rewarding pollinators. The stability of their ranks and IT was similar to that of those plants and pollinators that only appeared in two years (Table 4).

**Table 3:** Correlation of plants and pollinators between each pair of years.

		Plants			Pollinators		
		2008	2009	2010	2008	2009	2010
Rank	2007	0.53^**^	0.66^**^	0.62^**^	0.46^**^	0.35^*^	0.47**
IT		0.04	0.37**	0.16	0.60**	0.51**	0.23
Rank	2008		0.76^**^	0.63^**^		0.57^**^	0.38^*^
IT			0.25^*^	0.05		0.36^**^	0.27
Rank	2009			0.73^**^			0.38^*^
IT				0.41^**^			0.18

(* P<0.05; ** P<0.01). Rank is the position of plant and pollinator in maximum nestedness matric; Idiosyncratic temperature (IT) measures how a species' interaction pattern deviates from the position expected in a perfectly nested matrix.

**Table 4:** Correlation of plants and pollinators observed over all four years between each pair of years.

		Plants (n = 54)			Pollinators (n = 28)		
		2008	2009	2010	2008	2009	2010
Rank	2007	0.42^**^	0.63^**^	0.58^**^	0.58^**^	0.40*	0.41*
IT		0.06	0.30*	0.16	0.61**	0.46*	0.04
Rank	2008		0.82^**^	0.74^**^		0.47^**^	0.63^**^
IT			0.25	−0.04		0.42^*^	0.14
Rank	2009			0.79^**^			0.31
IT				0.45^**^			0.22

(* P<0.05; ** P<0.01).

The four-year data set permits us to compare pollination networks between consecutive and non-consecutive year-pairs, providing a more powerful examination of network dynamics than 3-year data. The average similarity index (mean of three pairs) of plants, pollinators and links was 0.68 vs 0.56, 0.30 vs 0.22, 0.10 vs 0.08 in the consecutive vs non-consecutive year-pairs respectively. The same indices from another four-year study of a scrub community in Greece (Petanidou *et al.* 2008), were 0.77 vs 0.70, 0.44 vs 0.41, 0.18 vs 0.17 respectively, were consistent with our finding of a higher similarity between consecutive years. The trend of less variation in plants, pollinators and their interactions between consecutive years was supported by stronger correlations in positions and link qualities of plants and pollinators. Rank positions and idiosyncratic temperatures were more similar between consecutive years than between non-consecutive years (Table 3), again suggesting the stability of the network is greater in consecutive years.

### Network turnover pattern

**Figure 2:**
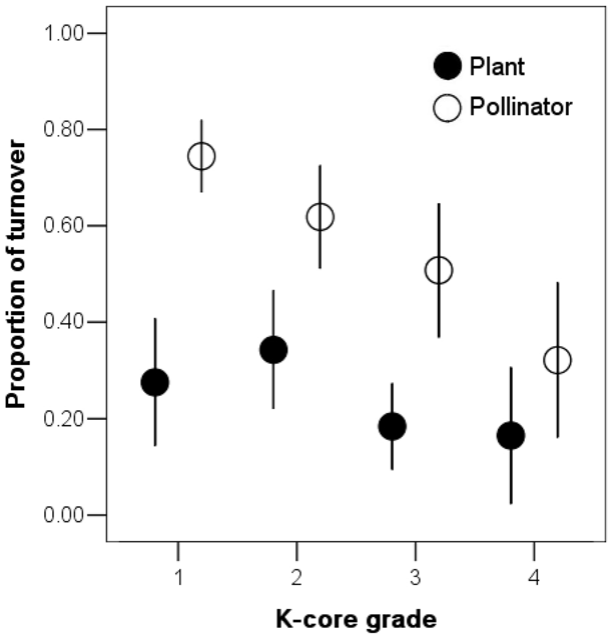
The turnover rates of plants and pollinators between k-core subgroups in each pair of years (Mean ± SD). (For subgroup standardization see Methods).

The turnover rate across *k*-core subgroups in both plants and pollinators can be calculated by comparing the turnover between each pair of years. The pattern of turnover among *k*-core subgroups showed that a highly connected generalist core was fairly stable across years for both plants and pollinators. Most transitions between *k*-core groups occurred in the periphery of the networks (Fig. 2). The average species turnover of inner grades was smaller than in periphery grades for both plants (17% vs 32%, G = 4.14, p = 0.04) and pollinators (43% vs 70%, G = 6.51, p = 0.01). For *k*-core grades, turnover rates were negatively related with the *k*-core number in both plants (Spearman correlation: N = 48, rho = −0.42, p<0.01) and pollinators (rho = −0.80, p<0.01). While the community composition of plants was less variable than that of pollinators in each year (see above), the inner group of pollinators obviously consisted of a more conservative core group. Species that were observed in any pair-year linked to almost their entire partner spectrum in single years. In six year-pair comparisons, identical species that were observed both years linked to over 90% of the partners of the annual networks. The plant and pollinator species that were observed across all four years were both linked to over 80% of the partners of the annual networks, suggesting that the relatively stable identical species could satisfy pollination services in the community even though networks experienced high turnover, assuming that these pollinators were equally effective at pollinating the plants. Each species in the networks had at least one link. However, only 7.1%±5.4% of the links of the annual network were contributed by the replaced species. This also indicated the peripheral position of the relative specialists, from another perspective.

Between inner and peripheral grades, only in 2009 and 2010, there were more hoverflies present in the inner group (G = 5.46, p = 0.02; G = 8.13, p<0.01). This implied that most functional groups were equally distributed in the inner-periphery pattern, although some functional groups fluctuated in certain years. The proportion of Diptera decreased from inner (56.2%±5.8%) to periphery (45.7%±5.7%), while the proportion of Hymenoptera did the opposite, increasing from 27.8%±6.1% to 30.7%±3.7%.

### Temporal variation of specialization

**Figure 3:**
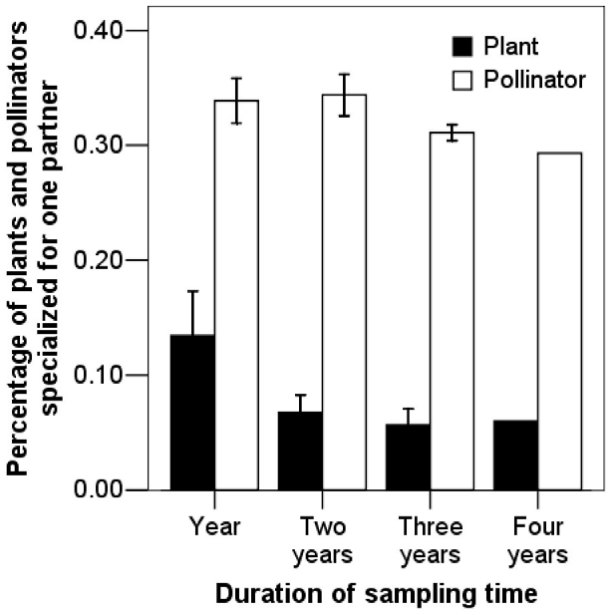
Variation of the specialist ratio over different sampling periods (Mean ± SD).

Across four years, most plants linked with more than one partner at both species level and pollinator functional group level (Table 5). However, plant species were relatively more specialized at the pollinator functional group level (Table 5). Across the full four-year dataset, plants linked to an average of 3.04±1.63 functional groups with 88 (66.2%) plant species linked to at least two functional groups in any observed year. Among these, 36 (27.1%) plant species tended to be generalists for the four consecutive years. However, 20 (15.0%) plant species were ecologically specialized to one functional group in all years, which was almost twice the number (11 plants) doing so at the species level. Among these, six plant species were functionally specialized to only bumblebees in the study community. Compared to plants, pollinators were more specialized (Table 5); 97 (29.3%) pollinators appeared to be specialists and were only observed in one year. Despite a higher turnover rate in pollinators and links, there were 17 (5.1%) pollinators specialized to one plant for more than one year. Focusing on annual networks, 13.5%±7.7% of plant species and 33.9%±3.9% of pollinator species were specialized. But levels of specialization of species decreased with increasing sampling effort (Fig. 3). Over the four years, only 6.0% of plants and 29.3% of pollinators were specialized to one partner in the network.

**Table 5:** Percentage of plants and pollinators according to the number of partner species linked.

	Plant	Plant*	Pollinator
Links to more than one partner across observed years	99 (74.4%)	88 (66.2%)	168 (50.8%)
Switch between links to one or several partners	23 (17.3%)	25 (18.8%)	49 (14.8%)
Links to one partner across observed years	11 (8.3%)	20 (15.0%)	115 (34.7%)

Percentages are taken over the total species pool (plants, 139; pollinators, 347 species). (* = pollinators at the functional level).

**Figure 4:**
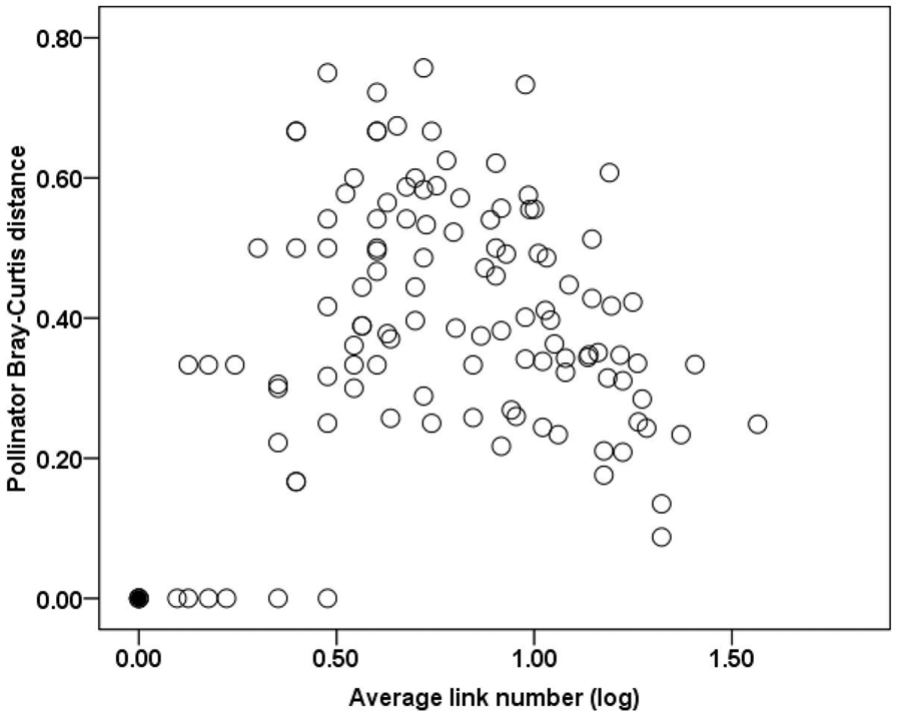
Relationship between plant link number and pollinator Bray-Curtis distance. The Bray-Curtis distance measures the range of variation in pollinator groups. (The black circle represents zero in three plant species that have same functional group partition).

At the functional level, both specialized and generalized plants experienced stable relationships with pollinators despite high species turnover. For the 107 plant species observed in at least two years, 104 (97.2% of 107) had at least one functional group remaining across years. No plant species experienced entirely different functional groups across years in this community. The proportion of identical functional groups across years in the total observed functional groups for each plant species was 69.1%±23.6% on average, indicating that most plant species had not only the same functional group across years, but also steady links to over half of the functional groups that were ever observed. In the annual networks, for each plant species 65.8%±38.5% of pollinator species belonged to identical functional groups that were observed in the next year. Furthermore, the average Bray-Curtis distance of pollinator composition variation for plant species between year pairs was not related to the average link levels of plants (Spearman rank correlation, β = −0.04, p = 0.69) (Fig. 4). Both ecologically specialized and generalized plants had relatively low variation in pollinator functional groups, suggesting that stable relationships with pollinators were established not only by specialized plants, but also by generalized plants. Variation in pollinator composition across years was relatively low in species of Apiaceae, Dipsacaceae, Asteraceae, *Pedicularis*, *Primula*, and *Potentilla*, but was relatively high in species of Gentianaceae, Brassicaceae and *Ligularia*.

## Discussion

Our four-year investigation of pollination networks in a biodiversity hotspot indicated that the networks were relatively stable even though species assemblages changed significantly. Plants and pollinators were consistent in their positions and link qualities in networks between years. Furthermore, inner groups comprising relatively ecologically generalized species were more stable than peripheral groups comprising relatively ecologically specialized species. The same species linked to almost the entire partner spectrum each year. Theoretical modeling predicts a high proportion of specialized pollination links in species-rich communities [9], a pattern not observed in this study of a biodiversity hotspot. Instead, both specialized and generalized plants experienced temporal stability of pollinator linkages.

Our results are compatible with those of several previous studies. As in other networks, there were more pollinator species than plant species each year and plant-pollinator interactions were nested [8], [17], [39]. Plants and pollinators were in stable rank positions while their link qualities (IT) were more changeable. Species with high IT have a higher chance of forming specialist-to-specialist links. But the variation in IT across years rearranged the links and specialist-specialist links did not stabilize even though they may be formed in certain years. Examination of an Arctic pollination network in day-to-day resolution [13] also suggested that certain specialist-specialist links could continue for a certain period, but newly emerged species preferentially linked to generalists, and this rearranged links. More stable relationships in plant ranks may result from the steady links between specialized plants and bumblebee pollinators in the study community. Thus, even though species composition fluctuated across years, the remaining specialized plants could remain at their original position in the network. Morphological constraint (e.g., accessibility of nectar) has been considered an important factor governing pollination network structure [40]. Bumblebees, for example, had relatively long proboscises enabling them to probe nectar from various flowers with different tube lengths, and this may explain the positive relationship in IT of these species between years in our community.

Our investigations indicate that plant-pollinator networks are relatively stable between years, consistent with previous studies [14]–[18]. A comparably high species turnover has also been observed in other ecosystems in the temperate zone [e.g. 41], [42]. High turnover rate restricts the formation of specialist-specialist links. However, we did observe tight interactions between bumblebees and plant genera including *Aconitum*, *Astragalus*, *Delphinium*, *Lotus*, *Pedicularis* and *Primula*. In a nested pattern, generalized species are more important than specialists for their resilience to local extinction, because the extinction of specialized species removes fewer links in the network leaving a greater number of species linked to their generalist partners [20]. Our *k*-core analysis indicates that the stabilized inner groups, which consists of generalized plants and pollinators, links most partners across years, suggesting stability of the nested structure. The stability of the generalized core groups also supports Memmott's [20] simulation model of extinction by field observation, emphasizing that generalists are more important to network stability, especially for the most generalized pollinators, such as the bumblebee fauna which link to nearly 50% of plant species. Our investigation indicates that bumblebees are core species providing pollination service in the alpine meadow.

The highly diverse pollination network studied here differs in several respects from networks studied in species-poor areas. First, the pollinator-plant ratio (average 1.48±0.09 for each year; 2.49 over all four years) in our community is much lower than that of other pollinator-plant networks [6], which generally have ratios above 4. Species richness has been considered to influence structural properties of a network [6], [43]. For example, as networks grow larger, there tend to be more asymmetric interactions in larger pollination networks compared to smaller ones [3]. We observed that some plant genera share the same pollinator functional group or even the same pollinator species [e.g. 44]. For example, *Bombus richardsi* was always the most generalized pollinator over the four years, and it was observed to link with over 50% of plant species in the community each year. These results support the prediction of strong pollinator competition in species-rich communities [26]. Second, the predicted high proportion of specialized pollinators in a species-rich community [9], [26] was not observed in our study. Only a small proportion of plant species were pollinated by a single pollinator species (13.5%±7.7%) and single pollinator functional group (20.4%±6.9%). Similarly, there was on average 13.0±7.1% of plant species pollinated by a single pollinator based on four surveys in North America [see 7]; in one community 54.0% of plant species were pollinated by one functional group [see 29]. Contrary to a null model that predicts a higher proportion of both generalized and specialized pollination links in species-rich than species-poor communities [9], our four-year data in this community showed that nearly 80% of plant species were pollinated by at least two pollinator functional groups. The proportion of specialized pollination links was not high in this species-rich community, consistent with two recent studies [16], [45]. The predicted increase of specialization of pollination systems during community succession was not observed along the chronosequence of a glacier foreland in southeastern Switzerland [16]. An investigation of flower supply and flower-visiting insects in 27 meadows in southern Germany indicated that the level of specialization did not significantly differ across the gradient of flower diversity [45]. Third, in our study Hymenoptera (bumblebees and other bees), rather than Diptera (flies), were highly generalized. Flies were observed to be more abundant in species and links than were bees in a sub-arctic alpine site in north Sweden [46] and in Andean meadows [47]. Dipteran species number (49.9%±5.2%) and link number (47.2%±5.1%) were predominant in our study, while Hymenopteran species contributed less, 29.5%±4.0% in species number and 38.1%±6.1% in link number. However, Hymenoptera linked to more plant species compared to Diptera (F = 2.36, p<0.01), indicating that Hymenoptera were more generalized than Diptera. Another investigation at the same site indicated that visits of bumblebees were more numerous than the total visits of all other pollinator groups [33].

High turnover rate in pollinator species does not necessarily imply high variation in pollination roles at a functional level. We found that plant species usually retain steady relationships to one or several functional groups over time, despite various levels of generalization. For example, the most generalized plant *Pleurospermum davidii* (Apiaceae) linked to 116 pollinator species across all 9 functional groups in four years. However, it had 4 functional groups consecutively, accounting for 88.4%±1.7% of the pollinators of the annual networks. These results suggest that generalized plants might experience stable evolutionary relationships with diverse functional groups. Different functional pollinator groups showed preferences for certain flower trait or traits combinations in the community [48]. Such stability and preferences of functional pollinator groups may contribute to the maintenance of diverse species in one community, although network compositions are highly changeable.

Our finding of the same pattern of network structures in all four years suggests that we have captured the ecologically important interactions, given that a transect sample procedure was used in 2007 but timed observation procedures were used from 2008 to 2010. Our binary data prevent us from considering the visit frequency of each link. However, from the perspective of species duration and link partner turnover, a binary network could represent the dynamic nature of pollination at the community level. In summary, by comparing pollination networks over four years, we found the structure of the pollination network to be stable, and the fluctuation in species composition mostly represented in the periphery of the networks, without changing network shape. The pollination network in this highly diverse community is robust and plants at different generalization levels experience similar variation in pollinator functional groups, although there is a great fluctuation in community species composition as observed at other sites [6], [19]. Our results also supported a recent 12-year butterfly plant network study, which suggested a separation between relatively stable species and sporadic species [49]. Our multi-year survey represents one of first studies on pollination networks in the biodiversity hotspot from China. Clearly, further study is needed if we are to understand how the generalized pollinators sustain diverse plant species in this alpine area. For example, more explicit data sets are needed to quantify the dynamics of pollination systems and explain how plants avoid reproductive interference in a highly generalized pollination system.

## Supporting Information

Supporting Information S1 Plant and pollinator species and their four-year links in the alpine community at a biodiversity hotspot, SW China. Data are presented as an interaction matrix, in which cells with ones indicate the interaction between a pair of species, and cells with zeros indicate no interaction. Data of four years are separately enclosed in four sheets of an Excel file.
(XLS)

## References

[1] Bascompte J, Jordano P, Melian CJ, Olesen JM (2003). The nested assembly of plant-animal mutualistic networks.. Proc Natl Acad Sci USA.

[2] Ollerton J, Johnson SD, Cranmer L, Kellie S (2003). The pollination ecology of an assemblage of grassland asclepiads in South Africa.. Ann Bot.

[3] Vazquez DP, Aizen MA (2004). Asymmetric specialization: a pervasive feature of plant-pollinator interaction.. Ecology.

[4] Petanidou T, Potts SG, Waser NM, Ollerton J (2006). Mutual use of resources in Mediterranean plant-pollinator communities: how specialized are pollination webs?. Plant-pollinator interaction: from specialization to generalization.

[5] Jordano P, Bascompte J, Olesen JM, Waser NM, Ollerton J (2006). The ecological consequences of complex topology and nested structure in pollination webs.. Plant-pollinator interaction: from specialization to generalization.

[6] Vazquez DP, Blüthgen N, Cagnolo L, Chacoff NP (2009). Uniting pattern and process in plant-animal mutualistic networks: a review.. Ann Bot.

[7] Waser NM, Chittka L, Price MV, Williams NM, Ollerton J (1996). Generalization in pollination systems, and why it matters.. Ecology.

[8] Dupont YL, Hansen DM, Olesen M (2003). Structure of a plant-flower-visitor network in the high-altitude sub-alpine desert of Tenerife, Canary Islands.. Ecography.

[9] Vazquez DP, Aizen MA, Waser NM, Ollerton J (2006). Community-wide patterns of specialization in plant-pollinator interactions reveal by null models.. Plant-pollinator interaction: from specialization to generalization.

[10] Basilio AM, Medan D, Torretta JP, Bartoloni NJ (2006). A year-long plant-animal mutualistic networks.. Austral Ecol.

[11] Herrera CM (1988). Variation in mutualisms: the spatiotemporal mosaic of a pollinator assemblage.. Biol J Linn Soc.

[12] Schemske DW, Horvitz CC (1989). Temporal variation in selection on a floral character.. Evolution.

[13] Olesen JM, Bascompte J, Elberling H, Jordano P (2008). Temporal dynamics in a pollination network.. Ecology.

[14] Petanidou T, Kallimanis AS, Tzanopoulos J, Sgardelis SP, Pantis J (2008). Long-term observation of a pollination network: fluctuation in species and interactions, relative invariance of network structure and implications for estimates of specialization.. Ecol Lett.

[15] Dupont YL, Padron B, Olesen JM, Petandiou T (2009). Spatio-temporal variation in the structure of pollination networks.. Oikos.

[16] Albrecht M, Riesen M, Schmid B (2010). Plant-pollinator network assembly along the chronosequence of a glacier foreland.. Oikos.

[17] Alarcón R, Waser NM, Ollerton J (2008). Year-to-year variation in the topology of a plant-pollinator interaction network.. Oikos.

[18] Burkle L, Irwin I (2009). The importance of interannual variation and bottom-up nitrogen enrichment for plant-pollinator networks.. Oikos.

[19] Burkle LA, Alarcón R (2011). The future of plant-pollinator diversity: understanding interaction networks across time, space, and global change.. Am J Bot.

[20] Memmott J, Waser NM, Price MV (2004). Tolerance of pollination networks to species extinctions.. Proc R Soc Lond B.

[21] Borgatti S, Everett MG, Freeman LC (2002). UCINET for Windows: software for social network analysis.

[22] Wuchty S, Almaas E (2005). Evolutionary cores of domain co-occurrence networks.. BMC Evol Biol.

[23] Myers N, Mittermeier RA, Mittermeier CG, Fonseca GAB, Kent J (2000). Biodiversity hotspots for conservation priorities.. Nature.

[24] Parrish JAD, Bazzaz FA (1979). Difference in pollination niche relationships in early and late successional plant communities.. Ecology.

[25] Olesen JM, Jordano P (2002). Geographic patterns in plant-pollinator mutualistic networks.. Ecology.

[26] Vamosi JC, Knight TM, Steets JA, Mazer S, Burd M (2006). Pollination decays in biodiversity hotspots.. Proc Natl Acad Sci USA.

[27] Pettersson MW (1991). Pollination by a guild of fluctuating moth populations: option for unspecialization in *Silene vulgaris*.. J Ecol.

[28] Armbruster WS, Fenster CB, Dudash MR (2000). Pollination “principles” revisited: specialization, pollination syndromes, and the evolution of flowers.. Det Norske Videnskapsacademi I. Matematisk Naturvidenskapelige Klasse Skrifter, Ny Serie.

[29] Fenster CB, Armbruster WS, Wilson P, Thomson JD, Dudash MR (2004). Pollination syndromes and floral specialization.. Ann Rev Ecol Evol Syst.

[30] Mayer C, Adler L, Armbruster WS, Dafni A, Eardley C (2011). Pollination ecology in the 21st century: key questions for future research.. J Pollinat Ecol.

[31] Dalsgaard B (2008). Pollination networks and functional specialization: a test using Lesser Antillean plant-hummingbird assemblages.. Oikos.

[32] Memmott J (1999). The structure of a plant-pollinator food web.. Ecol Lett.

[33] Gong YB, Huang SQ (2009). Floral symmetry: pollinator-mediated stabilizing selection on flower size in bilateral species.. Proc R Soc B.

[34] Guimãraes PR, Guimãraes P (2006). Improving the analyses of nestedness for large sets of matrices.. Environ Model Software.

[35] Almeida-Neto M, Guimaraes P, Guimaraes PR, Loyola R, Ulrich W (2008). A consistent metric for nestedness analysis in ecological systems: reconciling concept and measurement.. Oikos.

[36] Atmar W, Patterson BD (1993). The measure of order and disorder in the distribution of species in fragmented habitat.. Oecologia.

[37] Batagelj V, Zaversnik M (2002). Generalized Cores.. http://arxiv.org/abs/cs/0202039.

[38] Bray J, Curtis J (1957). An ordination of upland forest communities of southern Wisconsin.. Ecol Monogr.

[39] Petanidou T, Ellis WN (1993). Pollinating fauna of a phryganic ecosystem: composition and diversity.. Biodiv Lett.

[40] Stang M, Klinkhamer PGL, Waser NM, Stang I, van der Meijden E (2009). Size-specific interaction patterns and size matching in a plant-pollinator interaction web.. Ann Bot.

[41] Williams NM, Minckley RL, Silveira FA (2001). Variation in native bee faunas and its implications for detecting community change.. Conserv Ecol.

[42] Price MV, Waser NM, Irwin RE, Campbell DR, Brody AK (2005). Temporal and spatial variation in pollination of a montane herb: a seven-year study.. Ecology.

[43] Jordano P (1987). Patterns of mutualistic interactions in pollination and seed dispersal: connectance, dependence asymmetries, and coevolution.. Am Nat.

[44] Huang SQ, Fenster CB (2007). Absence of long-proboscid pollinators for long-corolla-tubed Himalayan *Pedicularis* species: implications for the evolution of corolla length.. Int J Plant Sci.

[45] Fründ J, Linsenmair KE, Blüthgen N (2010). Pollinator diversity and specialization in relation to flower diversity.. Oikos.

[46] Elberling H, Olesen M (1999). The structure of a high latitude plant-flower visitor system: the dominance of flies.. Ecography.

[47] Arroyo MTK, Primack R, Armesto J (1982). Community studies in pollination ecology in the high temperate Andes of central Chile. I. Pollination mechanisms and altitudinal variation.. Am J Bot.

[48] Gong YB, Huang SQ (2011). Temporal stablilty of pollinator preference in an alpine plant community and its implications for the evolution of floral traits.. Oecologia.

[49] Olesen JM, Stefanescu C, Traveset A (2011). Strong, long-term temporal dynamic of an ecological network.. Plos One.

